# Synthesis, molecular dynamics simulation and adsorption study of different pollutants on functionalized mesosilica

**DOI:** 10.1038/s41598-020-80566-w

**Published:** 2021-01-21

**Authors:** Rasool Pelalak, Roozbeh Soltani, Zahra Heidari, Rahime Eshaghi Malekshah, Mohammadreza Aallaei, Azam Marjani, Mashallah Rezakazemi, Saeed Shirazian

**Affiliations:** 1 Institute of Research and Development, Duy Tan University, Da Nang, 550000 Vietnam; 2 Faculty of Environmental and Chemical Engineering, Duy Tan University, Da Nang, 550000 Vietnam; 3 Department of Chemistry, Arak Branch, Islamic Azad University, Arak, Iran; 4 Chemical Engineering Faculty, Sahand University of Technology, P.O. Box 51335-1996, Sahand New Town, Tabriz, Iran; 5 Department of Chemistry, College of Science, Semnan University, Semnan, Iran; 6 Department of Chemistry, Faculty of Science, Imam Hossein University, Tehran, Iran; 7 Department for Management of Science and Technology Development, Ton Duc Thang University, Ho Chi Minh City, Vietnam; 8 Faculty of Applied Sciences, Ton Duc Thang University, Ho Chi Minh City, Vietnam; 9 Faculty of Chemical and Materials Engineering, Shahrood University of Technology, Shahrood, Iran; 10 Laboratory of Computational Modeling of Drugs, South Ural State University, 76 Lenin Prospekt, Chelyabinsk, Russia 454080

**Keywords:** Biochemistry, Chemistry, Engineering, Mathematics and computing

## Abstract

Experimental and computational works were carried out on a new type of mesoporous silica. In the experimental section, functionalized hollow mesosilica spheres were prepared via a facile technique and then evaluated using some analytical techniques (FESEM, TEM, L-XRD, FTIR, BET-BJH, and TGA). The obtained results revealed that the synthesized material had hollow structure with a diamino-grafted porous shell. The molecular separation of crystal Violet (CV) and neutral Red (NR) dyes from water were investigated by adsorption process using the synthesized powder. Influence of adsorbent loading was evaluated as adsorption ability and dyes removal efficiency. Also, the obtained modeling results revealed appropriate fitting of data with non-linear Langmuir model. The theoretical studies were employed to study the adsorption and removal mechanism of cationic (CV and NR) and anionic (orange II (OII)) dyes using molecular dynamics calculations. Moreover, the simulation outcomes provided valuable information about quantum chemical properties including the HOMO–LUMO maps, chemical reactivity, global softness (σ) and hardness (η) for silica-linker-water-dyes components.

## Introduction

Reactive dyes and pigments are being extensively employed in industrial activites like textile, food, pharma, and have been recognized as a source of water pollutions^1–4^. In recent years, large amount of dyes are being released into the surface and groundwater causing harm to aquatic organisms, ecological systems and human health^4–7^. Among various synthetic dyes being used worlwide, crystal violet (CV), neutral red (NR), and orange II (OII) are cationic and anionic dyes, which are largely utillized in the industrial activities^8^. Toxic effects of dyes at specific amount and their mutagenesis, cancer-causing and carcinogenic effects has been reported widely^9–12^. The presence of dyes in the effeluent of wastewater treatment plants have been stated by several reports. Several treatment methods have been proposed and used for decontamination of wastewater including biological, sedimentation, adsorption, membrane, and filtration^13–17^.

Adsorption as a highly efficient and costly separation/purification method has proved its ability for dye removal from aquatic solutions^18,19,20,21^ and numerous adsorbents such as silica, zeolite, carbone based materials, ashes, and mineral clays have been extensively sythnesized and utilliezed in pollutants elimination from aqueous media^22,23^. One of the attractive classes of adsorbents is mesoporous silica (MPS) materials as inorganic polymers, which are superior candidates for many environmental processes due to their superior properties such as well-ordered pore systems and relatively inexpensive starting materials as well as high surface area^24–26^. A lot of attention has recently been focused on MPS for dye adsorption from wastewater^27–30^. Also, recent developments of the in vivo toxicity, bio-distribution and clearance of mesoporous materials in animal models have shown excellent long-term applications^31^. Surface modification or functionalizing of MPSs with some organic functional groups extremely improves the physical characteristics of MPSs like enhancing the available area and adsorption ability due to better interaction between the pollutant and adsorbent^26,28,32,33^. MPS functionalized with amino group with improved properties such as enhancement in surface area and also adsorption performance have been used as effective adsorbent for wastewater treatment approaches^15,26,34–36^.

Beside the experimental works on development of MPSs, computational studies can be useful to understand the interaction at molecular level, and manipulate the functional groups for preparation of advanced porous materials for environmental applications. Molecular dynamics simulation is a great modelling tool which provides important understandings in molecular scale about adsorption process, interactions and transport properties^26,37^. Furthermore, usefule information about pollutant decomposition in atomic behavior can be provided by molecular dynamics computations.

In the current investigation, a molecular-level computational approach is utilized to understand the dye adsorption on the surface of MPS. Both experimental and computational works have been carried out. In the experiments, functionalization of MPS with amino group were investigated by one-pot synthesis method. Diamine-functionalized hollow mesosilica spheres (MPS-linker) adsorbent was fabricated and characterized with different tools, e.g. FESEM, XRD, FTIR, N_2_ adsorption–desorption and TEM. Adsorption ability of MPS-linker adsorbent was evaluated in adsorption of CV and NR as cationic dyes and OII as anionic dye through comprehensive experimental and modeling investigations. In the modeling section, the molecular dynamic simulations were performed. The results of simulations provide very valuable information about gap energies, HOMO, LUMO, hardness, and reactivity of the interactions among dyes and MPS.

## Materials and methods

### Materials

Diamino silane (1,4-Bis[3-(trimethoxysilyl)-propyl]ethylenediamine (DA) 97%) as coupling agent, tetraethyl orthosilicate (TEOS, ≥ 99.0%) as precursor and hexadecyltrimethylammonium bromide (CTAB) surfactant template were obtained from Sigma-Aldrich. Ultrapure ethanol and acetone were obtained from Merck, and purified water was used in all experiments.

### Synthesis procedure of adsorbent

One-pot procedure proposed by Soltani et al. was used for synthesis of MPS-linker adsorbent, with some modifications^28,35,36^. In short, predetermined amount of CTAB and ammonia solution as a template, were added to purified water. The obtained solution was mixed at ambient temperature for 20 min. In the next step, a required amount of DA as functionalization material was added to the solution and sonicated for 5 min. Another 5 min sonication were applied to the mixture after adding 42.5 mL of TEOS, as silica source. The procedure was continued by magnetically stirring for 180 min. After that, the bottle was placed in the oven and kept for 12 h at 343 K. The obtained mixture was rinsed several times with deionized water, ethanol, and acetone then dried at 348 K overnight. The final product was refluxed overnight in order to eliminate CTAB (the reflux solution was ethanol/HCl 12 N 100:1). After cooling, the obtained mixture was sonicated for 5 min, then filtered and rinsed with ethanol and deionized water. At the end, the samples dried at 343 K for 24 h.

### Simulations

The Materials Studio software was used for molecular quantum calculations through DMol3 module as reported in our previous work^26^. Prior to the computational calculations, the Hyperchem software was employed for initial optimizating the geometry of MPS-linker, water and dyes (CV, NR and OII). After that, the DMol3 module was employed for geometry and energy optimization, HOMO and LUMO energy levels, as well as COMSO sigma profile of the components. The simulated optimized structures were used for predicting the preferential adsorption of dyes on MPS-linker via adsorption locator and Forcite modules. A combination of MPS-linker/water/dye was selected for each run and the potential of their interactions were evaluated. In every simulation, a mixture of two molecules of dyes (NR or CV or OII), four molecules of water and one molecule of adsorbent were used to calculate the low energy configurations and energy distribution of the mixture.

## Results and discussion

### Characterization of adsorbent

**Figure 1:**
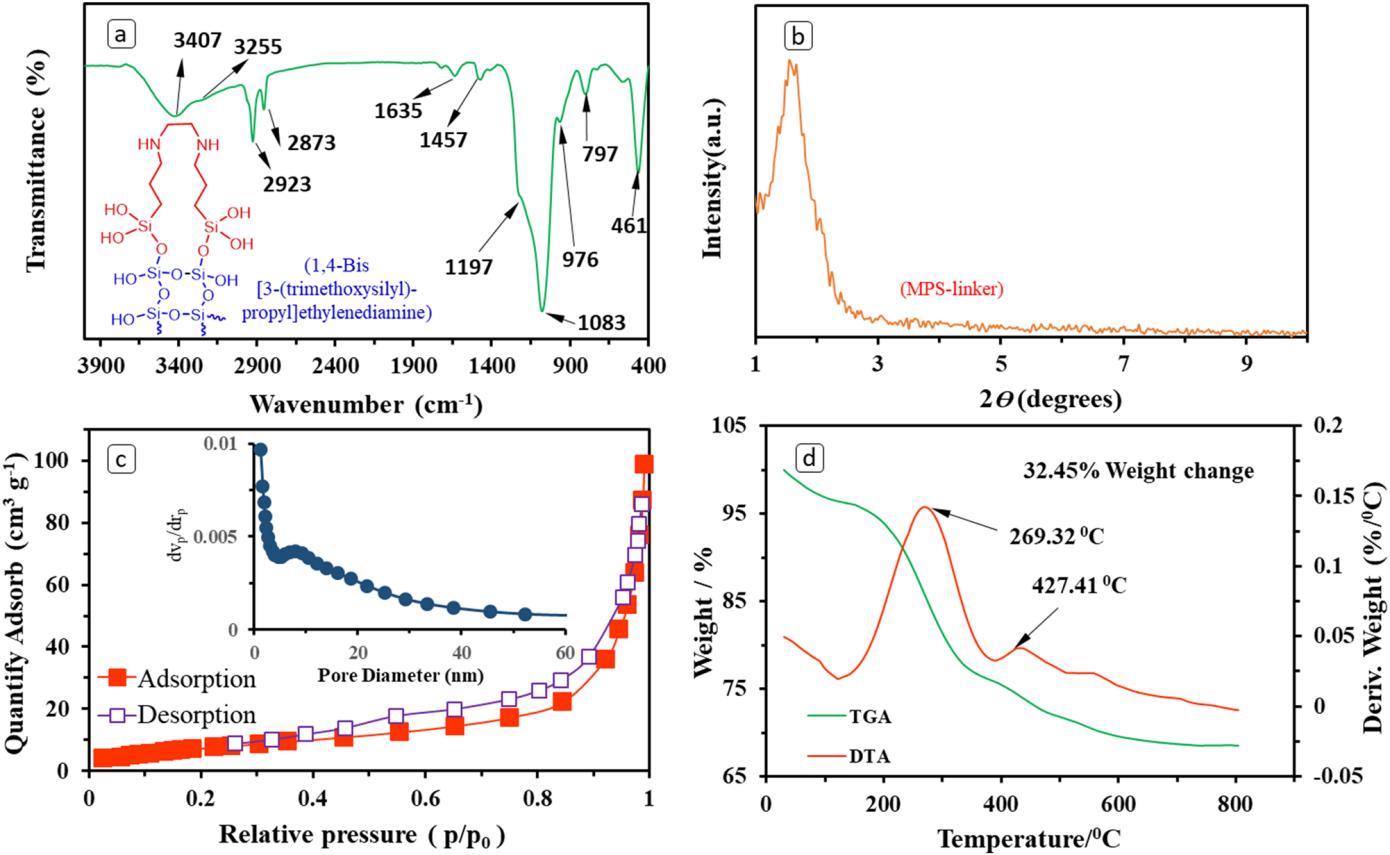
FTIR spectrum (**a**), L-XRD pattern (**b**), N_2_ adsorption–desorption isotherms (**c**), and TGA/DTA results (**d**) for the synthesized adsorbent.

For characterizing surface functional groups of the produced adsorbent, the Fourier transform infrared spectroscopy (FTIR, Tensor 27, Bruker, Germany) with wavelength range of 400–4000 cm^−1^ was applied using the KBr pellet technique. The obtained FTIR spectrum for the syntehsized adsorbent is presented in Fig. 1a. Also, the structure of MPS-linker is shown in Fig. 1a. The successful structure and composition of MPS-linker bearing diamino groups were observed by the obtained results^35,36,38,39^. The absorption bands around 461, 797, 1083 and 1197 cm^−1^ are corresponded to bending mode, symmetric and asymmetric bending vibrations of the Si–O–Si^6^. The appearing band at 976 cm^−1^ is assigned to stretching mode of Si–OH. Also, the band at 1457 cm^−1^ is related to the C–H bending vibration mode. The recorded band at 1635 cm^−1^ can be related to bending mode of –OH of the adsorbed H_2_O onto the surface^39^. The detected band around 2873–2923 cm^−1^ can be attributed to the C–H stretching vibrations of the methylene groups in the DA structure^40^. The band located around 3255 cm^−1^ is related to the –OH stretching vibration of adsorbed water and silanol groups^35^. The observed absorption band around 3407 cm^−1^ can be associated to –NH and –NH_2_– stretching vibration modes.

The crystalline structure of synthesized MPS-linker adsorbent was evaluated with the low-angle XRD (L-XRD) analyzer. The Siemens D5000 diffractometer XRD (Germany, scanning rate = 0.05° min^−1^) was employed in 2θ = 0.8–10.0°. The obtained L-XRD result of MPS-linker is shown in Fig. 1b. As can be seen, the first peak observed at 2θ = 1.0–2.5° is a relatively sharp peak attributed to the semi-long-range ordered hexagonally arranged mesostructure of the silica^15,28^. Convenience in accessibility of adsorption sites can lead to overcome the mass transfer problems which overally improve the process efficiency^15,25,35,36^.

The surface properties were achieved by N_2_ adsorption–desorption analysis, carried out at 77.3 K by using a Belsorp-mini II (Japan) and the obtained results are presented in Fig. 1c. As can be seen, the MPS-linker showed a combination of type IV(a) and V isotherms with a H3-hysteresis loop, according to the *IUPAC* assortment^27,41^. Also, the pore size distribution obtained by BJH model revealed that the size of pores can be lower and higher than 2 nm, indicating a bimodal micro-mesoporous structure for MPS-linker (the inset of Fig. 1c). The total pore volume (V_Tot_) and specific surface area (S_BET_) were around 0.15 cm^3^/g and 31 m^2^/g, respectively. Also, the diameter of spheres, calculated by *Nahamin Pardazan Asia* software, were in the range of 1–4 µm.

Thermogravimetric analysis (TGA) was performed to assess the successful surface modification (Fig. 1d). This analysis was achieved under active argon flow in a thermal analyzer (SDT Q600, USA) in range between 30 and 800 °C and a heating rate of 20 °C min^−1^. Figure 1d represents the thermal stability of prepared MPS-linker adsorbent. As can be inferred, a weight loss occurred below 200 °C was occured which can be due to the physisorbed water removal from the samples surface. The next weight reduction of MPS-linker adsorbent is observed above 300 °C which is primarily due to the destruction of the linker (NH_2_-silane molecules) attached to the MPS-linker surface. TGA results indicated that about 32.45% weight loss was found for the as-prepared MPS-linker adsorbent and it can be said that the synthesized samples were stable up to 435 °C in an active argon flow atmosphere.

**Figure 2:**
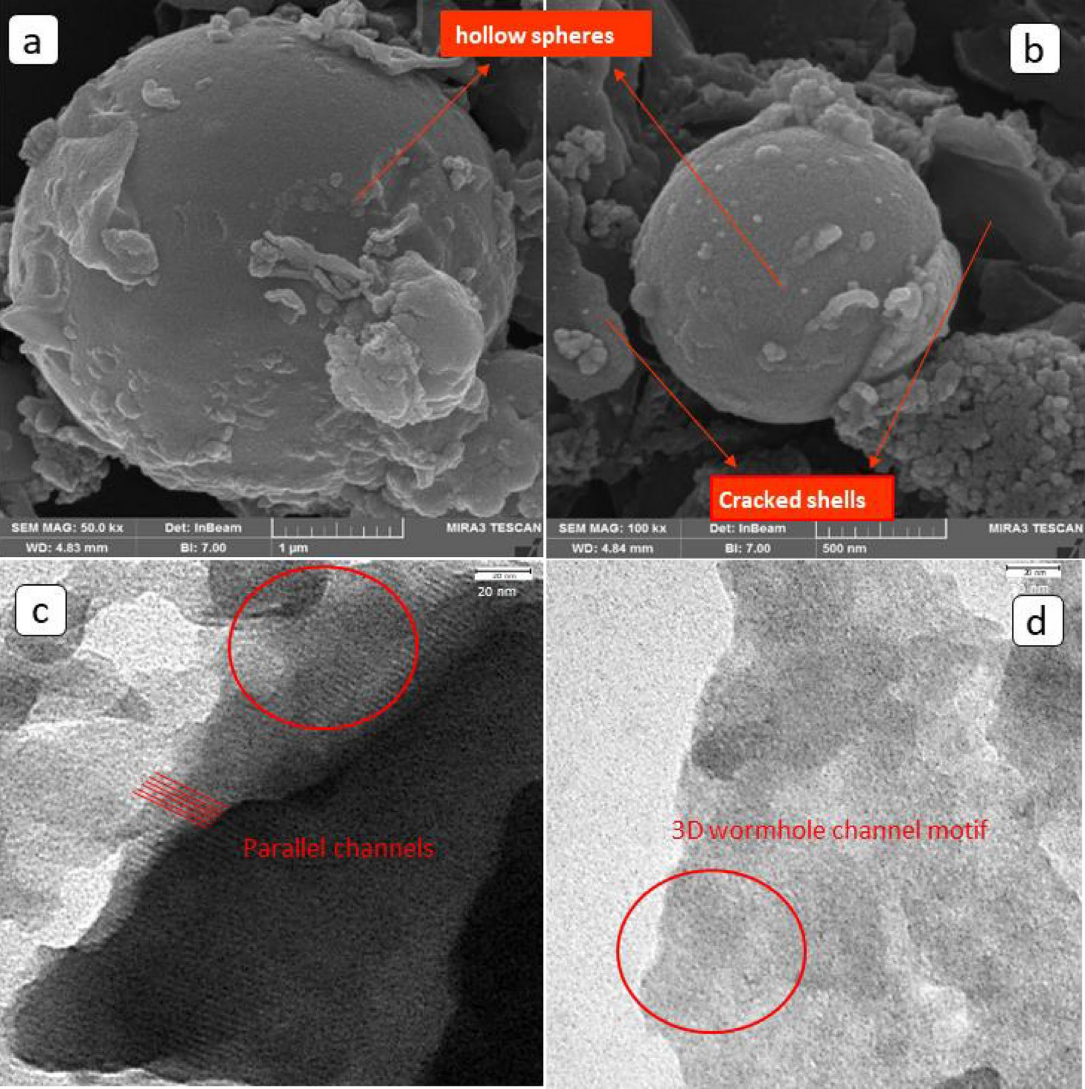
FESEM images (**a** and **b**), and TEM images (**c** and **d**) of the synthesized adsorbent.

The field emission scanning electron microscope (FESEM) (Mira 3-XMU microscope) was utilized to investigate the surface morphology of synthesized adsorbent. The obtained FESEM micrographs are presented in Fig. 2a,b. The spherical morphology of the MPS-linker could be obviously observed. The hollow structure of the asprepared adsorbent can be inferred from cracked shells detected in the image which is depicted in Fig. 2b. In order to investigate the structural and morphorogical properties of the synthesized material, the transmission electron microscopy (TEM) was accomplished. The TEM images of the adsorbent structure are shown in Fig. 2c,d. As indicated, MPS-linker particles possess both ordered and interconnected wormhole pore structure. Parallel arrays of ordered mesochannels as well as wormhole channel motifs could be clearly seen in Fig. 2c,d. Wormhole pore structure can effectively improves the mass transfer and removes the diffusion barriers.

### Experimental results

#### Investigating the impact of MPS-linker dosage on adsorption process

**Figure 3:**
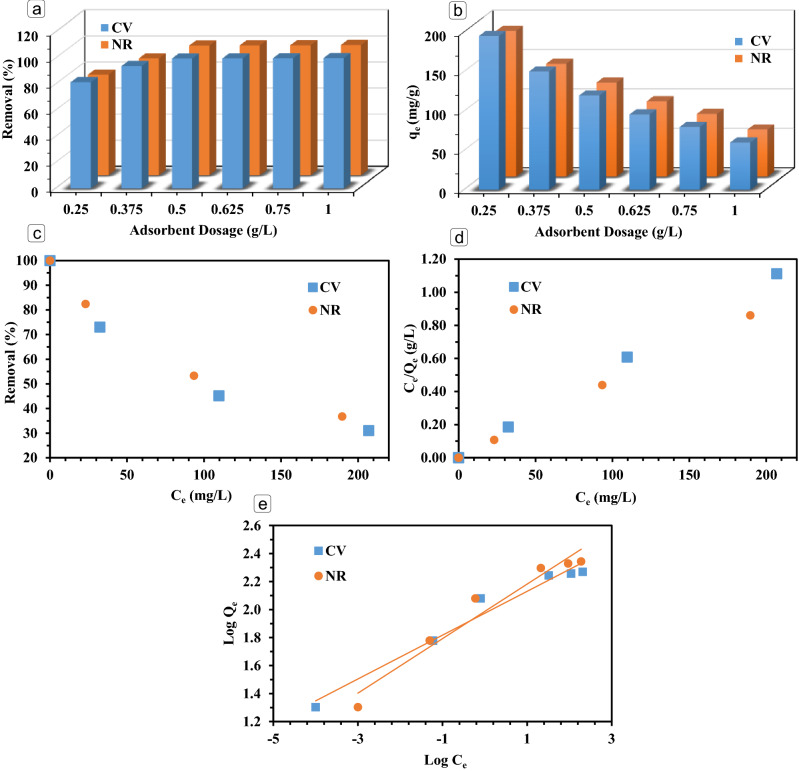
Impact of MPS-linker adsorbent loading on the CV and NR removal efficiency (**a**) and adsorption capacities (**b**), equilibrium isotherms (**c**), Langmuir (**d**) and Freundlich (**e**) models for CV and NR dyes adsorption on the synthesized MPS-linker adsorbent.

One of the significant factors on the adsorption efficiency is the amount of adsorbent loading. In this research, the effect of adsorbent dose on the removal efficiency of NR and CV was monitored, while the adsorbent dosages varried betweeen 0.25 and 1.0 g/L and other operating conditions were kept constant^35^. As can be seen in Fig. 3a,b the NR and CV removal were enhanced by increasing the MPS-linker dosage from 0.25 to 0.5 g/L, as expected^35^. The reason of this increment can be owing to the improvement in the availablilivy of adsorption sites of fabricated MPS-linker, by increasing the adsorbent loading. On the contrary, further increasing in the adsorbent loading (0.5–1 g/L) did not change the removal efficiency very significantly. Therefore, it can be revealed that the optimum dosage of MPS-linker is 0.5 g/L, and in all other tests were done at this value. In the case of adsorption capacity of MPS-linker, different trend was observed by increasing the adsorption dosage. As it can be seen in Fig. 3b, increasing the MPS-linker dosage reduced the adsorption capacity (q_e_) in both CV and NR dyes. It can be said that by increasing the adsorbent in a constant initial concentration of CV and NR dyes as pollutant molecules, provided a low concentration gradiant. This led to a negative influence on the pollutant attraction to the surface of MPS-linker.

#### Isotherm studies

The study of adsorption isotherms provide a deeper insight about adsorption capacity, adsorption mechanism between the pollutant and the adsorbent and the surface properties of MPS-linker. In this research, the behavior of CV and NR molecules adsorption on the prepared MPS-linker adsorbent were investigated using Langmuir and Freundlich isotherm models. According to Eqs. (1) and (2), the linear forms of Langmuir and Freundlich models can be achieved, respectively^35^:

1 $$\frac{{{\text{C}}_{{\text{e}}} }}{{{\text{q}}_{{\text{e}}} }} = \frac{{{\text{C}}_{{\text{e}}} }}{{{\text{q}}_{{{\text{max}}}} }} + \frac{1}{{{\text{q}}_{{{\text{max}}}} {\text{ K}}_{{\text{L}}} }}$$

2 $${\text{logq}}_{{\text{e}}} = {\text{logK}}_{{\text{f}}} + \frac{1}{{\text{n}}}{\text{logC}}_{{\text{e}}}$$

In these equations, q_max_ is the maximum adsorption capacity (mg/g), K_L_ is the constant value of the Langmuir model (L/mg). Also, K_F_ and n present the constants in the Freundlich model. The results of adsorption isotherms for NR and CV adsorption on the synthesized MPS-linker adsorbent are shown in Fig. 3c–e. The adsorption isotherm parameter values are as follow: for CV: K_L_ = 1.2, K_F_ = 94.2 and n = 6.4; for NR: K_L_ = 1.0, K_F_ = 97.1 and n = 5.1. The linear regression coefficient (R^2^) for Langmuir and Freundlich models were obtained as 0.999 and 0.960 for CV, and 0.999 and 0.938 for NR dyes, respectively. Therefore, it can be revealed that Langmuir isotherm model with higher R^2^ more properly described the adsorption behavior of both NR and CV dyes^35^.

A comparison of the adsorption capacity of NR and CV by the MPS-linker in this study with those stated in the literature is summarized in Table 1. It can be confirmed from the results that the synthesized MPS-linker adsorbent had almost the highest adsorption capacity for NR and CV dyes compared to other used adsorbents. This can be due to the presence of rich adsorption sites such as amine and hydroxyl groups, which can significantly provide a linkage between the MPS-linker surface and the NR and CV molecules^35^.

**Table 1:** Comparison of the capacity of adsorption for MPS-linker and other similar adsorbents (according to the Langmuir model).

Adsorbent	q_max_ for CV	q_max_ for NR	R^2^	References
MPS-linker	185	222	0.999	This work
Nanoporous carbon	68.97	–	0.988	^42^
Fe_3_O_4_/Chitosan/Glutaraldehyde nanocomposites	105.467	–	0.996	^43^
Modified kaolin nanoparticles	96.10	–	0.992	^44^
Gum arabic-cl-poly(acrylamide) nanohydrogel	90.90	–	0.974	^45^
Raw Tunisian Smectite Clay	86.54	–	0.988	^46^
Magnetic hollow spheres (Fe_2_O_3_)	–	105	0.992	^47^
Halloysite nanotubes	–	54.85	0.999	^48^
Typha orientalis carbon – MnCl_2_	–	199.10	0.8714	^49^
Typha orientalis carbon – Mn(NO_3_)_2_	–	198.37	0.9233	^49^
Boron nitride nanoparticle	–	26.20	0.995	^50^
Peanut husk	–	37.46	0.993	^51^

### Modeling results

#### Adsorption capacity analysis

**Figure 4:**
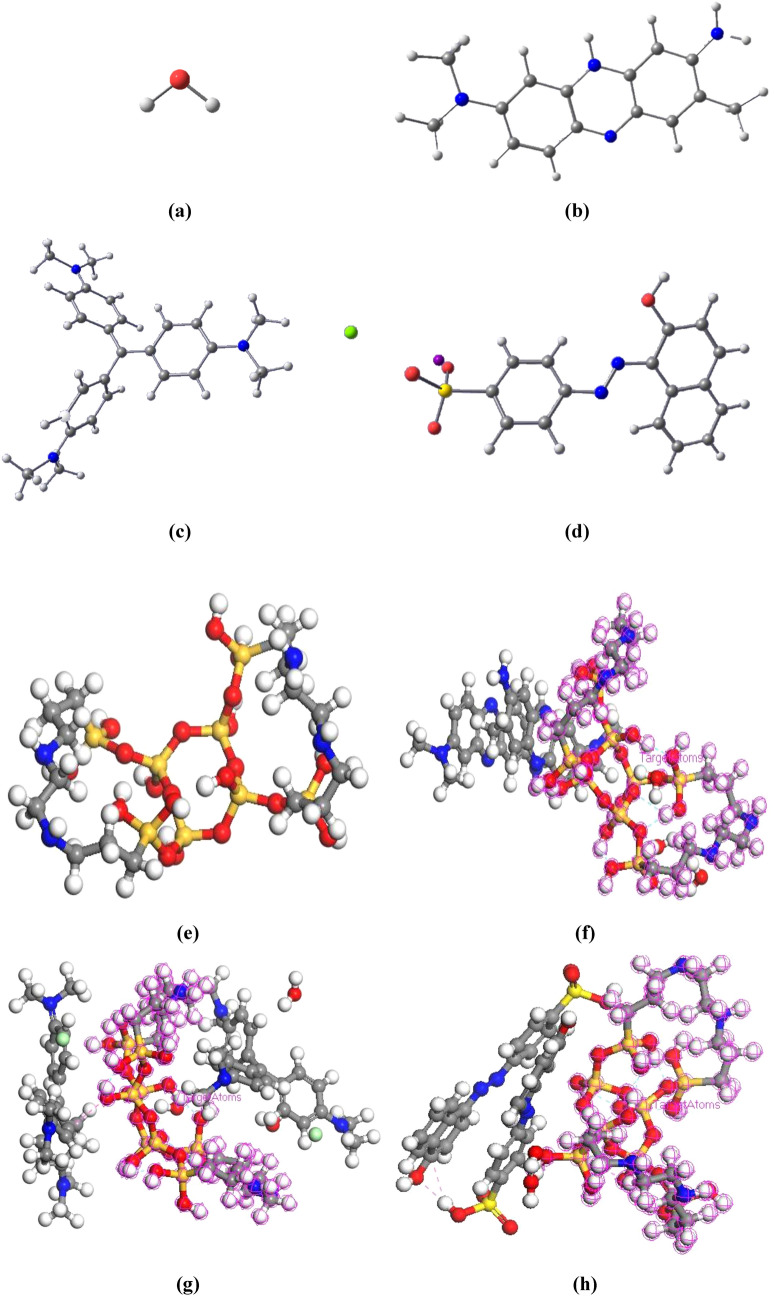
The optimized structures for water (**a**), NR (**b**), CV (**c**), OII (**d**), MPS-linker (**e**), MPS-linker-water-NR (**f**), MPS-linker-water-CV (**g**) and MPS-linker-water-OII (**h**).

The molecular dynamic simulations were investigated for adsorption of cationic dyes (CV and NR) on MPS-linker. Moreover, in this section the anionic dye adsorption ability of MPS-linker was studied for the orange II (OII) adsorption and the results were compared with the cationic dyes. In order to obtain the suitable position and energies of compounds, the structure of water, CV, NR, OII and MPS-linker were placed in the cell volume and optimized by Dmol3 module in Materials Studio software. The obtained results for the titled components as well as MPS-linker in the presence of water and dyes are shown in Fig. 4a–h. Based on the results, the total energy values of water, NR, CV, OII and MPS-linker were observed at − 75.91, − 793.49, − 1583.87, − 1415.98 and − 4653.41 kcal/mol after optimization, respectively. In addition, the calculated values of adsorption energy were obtained about − 113.92, − 247.00 and − 166.01 kcal/mol for MPS-linker-water-NR, MPS-linker-water-CV and MPS-linker-water-OII, respectively. The results of adsorption energy analysis indicated more negative adsorption energy value of compounds which represent trapping mechanism of dyes on the hollow mesosilica spheres. The adsorption energy of MPS-linker-water-NR was less negative than MPS-linker-water-CV and MPS-linker-water-OII. As a result, to remove NR dye using a chemical method, MPS-linker was less active in comparison with CV dye, meanwhile, to remove CV dye using a chemical method, MPS-linker was more active.

### Molecular calculations

**Figure 5:**
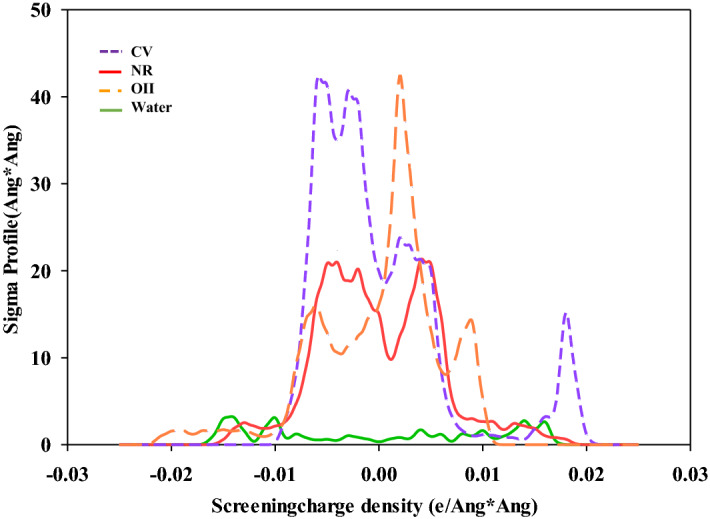
The obtained Sigma profile for NR, CV, OII dyes and water molecules.

In order to predict the distributions of surface polarity and molecular geometry of the compounds, Eckert et al.^52^ established the analyses of quantum chemical through a statistical mechanics. Here, in order to characterize the probability distribution of charge density and the number of surface area fragments the Sigma (*σ*) profiles were obtained for all titled components. To obtain *σ*-profiles, quantum mechanical calculations were applied and the results are shown in Fig. 5. The *σ*-profile for the nonpolar bonding of water was located on − 0.0084 e/Å^2^ < σ <  + 0.0084 e/Å^2^^53^. The *σ*-surface appeared at + 0.015 e/Å^2^ can be related to the lone-pair electrons on oxygen elements in water. In addition, the peaks around − 0.015 e/Å^2^ were attributed to the polar hydrogen. The observed peaks in symmetric regions, denoted the durable hydrogen bond between water and other molecules^54^. The *σ*–profile of the non-H-bonding for NR dye demonstrated sharp peaks in range of 0– + 0.015 e/Å^2^ which were related to the hydrogen elements in the methyl groups. Also, *σ*-profiles of the carbon of methyl groups, or the non–polar nitrogen groups of CV dye were detected between 0 and + 0.01 e/Å^55^. Furthermore, the obtained sigma profile of carbon atoms and the π-faced in the C rings were presented at about 0.0035 e/Å^56^. In Fig. 5, the strong peak detected at about 0.008 e/Å^2^ was related to the lone-pair electrons of oxygen in OII dye. In addition, very high intensity peak for the nitrogen in the OII structure was displayed about 0.0020 e/Å^2^.

**Figure 6:**
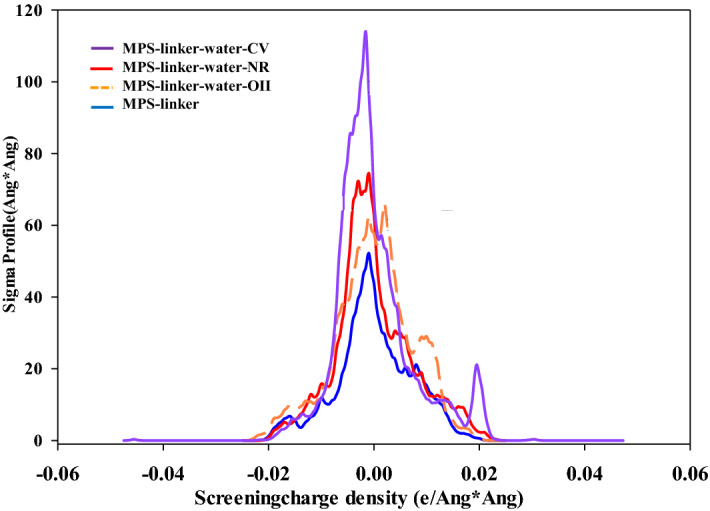
The obtained Sigma profile for MPS-linker-water-NR, MPS-linker-water-CV, MPS-linker-water-OII and MPS-linker.

The observed peak of MPS-linker molecule around + 0.008 e/Å^2^ can be related to a pair of valence electrons on oxygen (Fig. 6). The presented peak at − 0.009 e/Å^2^ was attributed to the hydrogen atoms. Some low intensity peaks were detected around + 0.005, which can be assigned to the electron-withdrawing fragments of the N groups in the MPS-linker structure. The high peaks between 0.010 and 0.018 e/Å^2^ in MPS-linker-water-NR were associated to the cyclic nitrogen of adsorbed NR dye. Also, the obtained peaks of the sigma profile for MPS-linker-water-CV molecule observed in the region between + 0.010 and + 0.021 e/Å^2^ were attributed to the N groups, and the presence of chlorine molecule. Finally, the observed peaks of the sigma profile for MPS-linker-water-OII molecule was detected around 0.0018 e/Å^2^ which can be related to the nitrogen groups.

#### Frontier molecular orbital analysis of compounds

**Figure 7:**
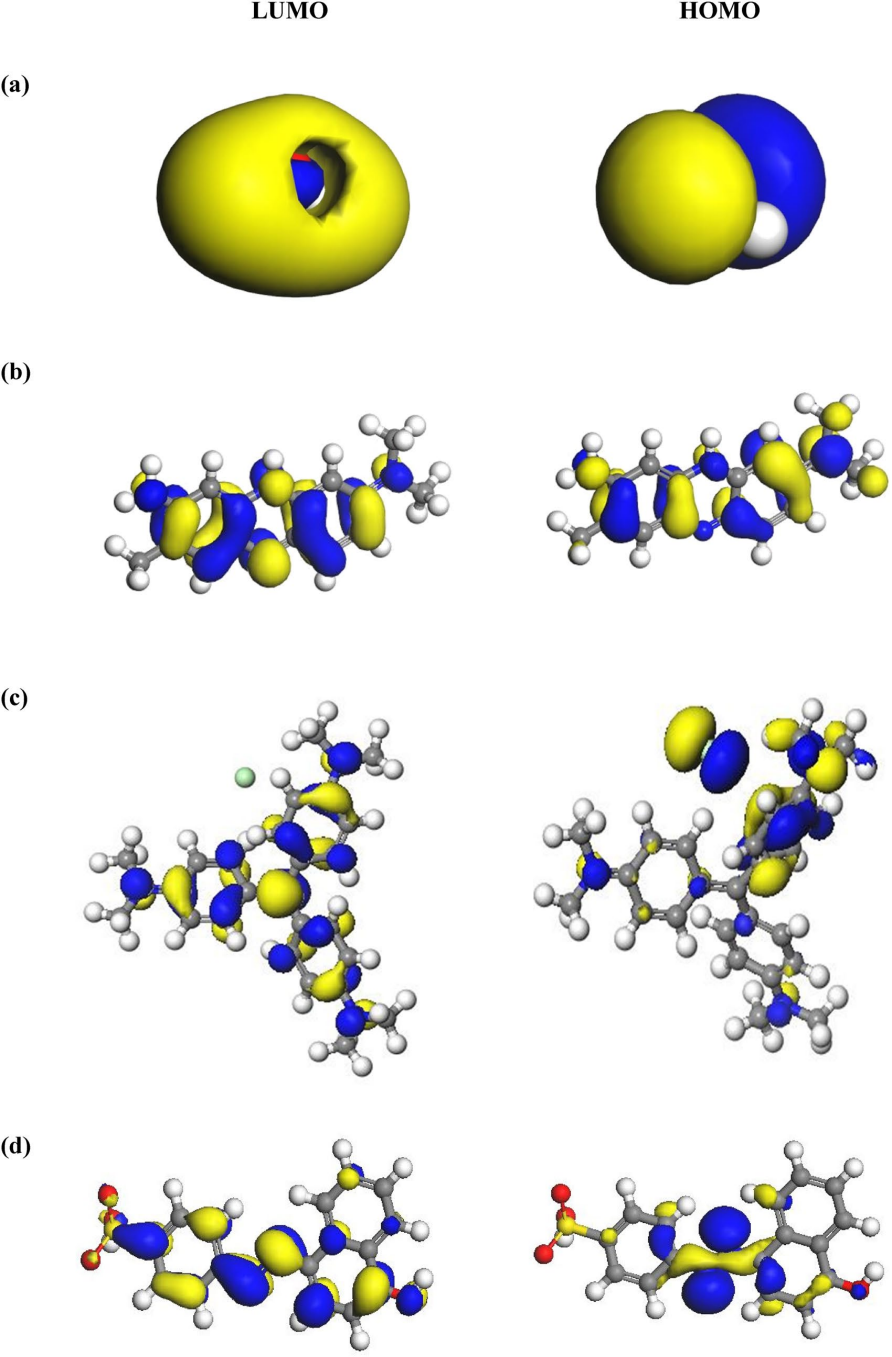
The HOMO and LUMO energy levels for water (**a**), NR (**b**), CV (**c**) and OII (**d**) dye molecules.

The results of the highest occupied and the lowest unoccupied molecular orbitals (HOMOs and LUMOs, respectively) for differernt structures are presented in Fig. 7a–d. Some quantum chemical parameters can be obtained using the energy gap (E_gap_) between frontier molecular orbitals (FMOs) such as electronegativity, electrophilicity index, the polarizability, global hardness and softness. The HOMO energy level of water, NR, CV and OII dyes were about − 0.2573, − 0.1743, − 0.1744 and − 5.3570 eV, respectively, meanwhile, the calculated LUMO energy level for water, NR, CV and OII dyes were positioned at about + 0.0567, − 0.1073, − 0.1215 and − 3.7070 eV, respectively. As shown in Fig. 7a–d, the HOMO energy levels of water were principally localized on the oxygen atom, whereas the LUMO energy levels were placed largely on hydrogen atom. Also, the HOMO and LUMOs levels of NR dye were mostly placed on the benzene ring (the N element) and on the hydrogen atom. In addition, in the electron charge distributions of HOMOs in CV dye were placed on the aromatic rings, nitrogen, and chlorine elements, whereas LUMO maps were distributed on the charge distribution of hydrogen atom. The HOMOs of OII dye were largely distributed on N atom as well as O atom of hydroxyl, while the LUMOs were generally localized on carbones of benzene ring and S element of dye.

**Figure 8:**
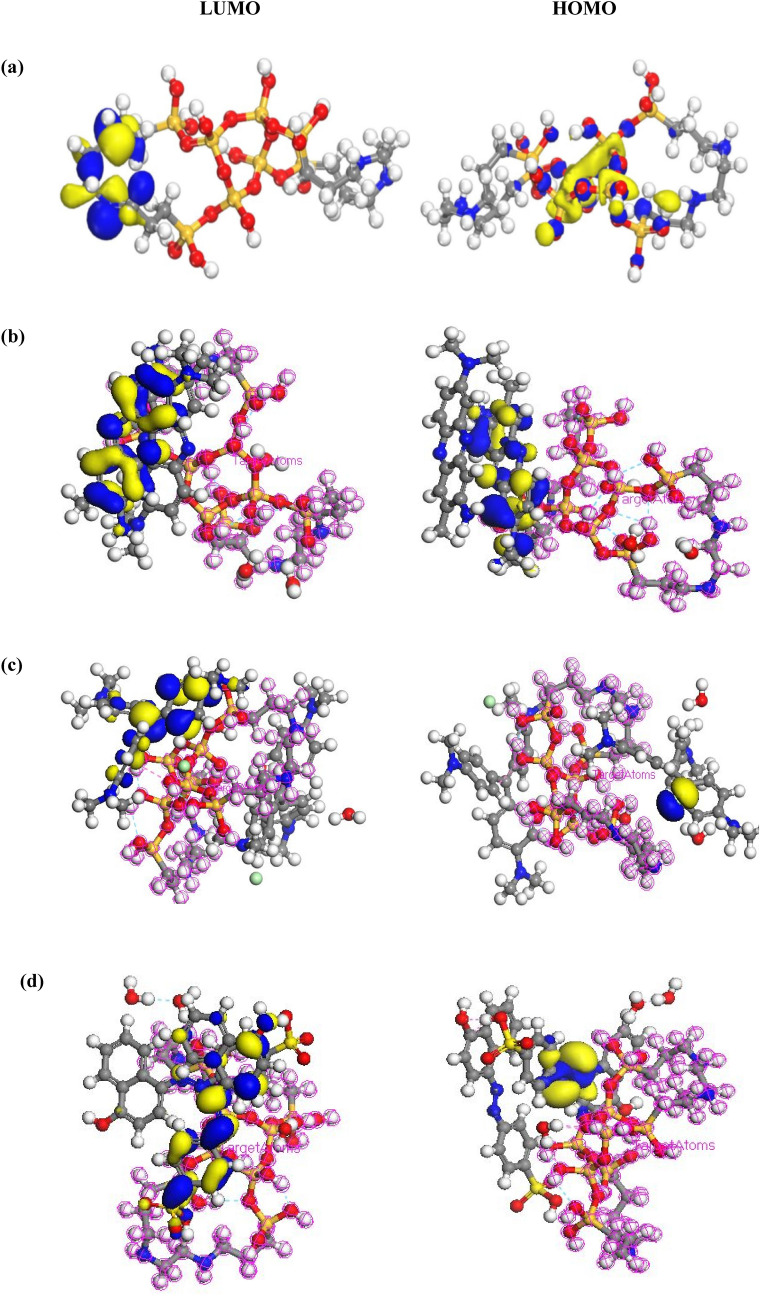
The HOMO and LUMO energy levels for MPS-linker (**a**), MPS-linker-water-NR (**b**), MPS-linker-water-CV (**c**) and MPS-linker-water-OII (**d**).

The HOMO and LUMO maps of MPS-linker were measured at about − 10.1540 and + 3.5270 eV, respectively. The HOMOs were localized on N atoms of linker, while the LUMOs were mostly distributed on O and Si atoms of silica (Fig. 8a). Moreover, the energy values of + 7.4830 and − 0.8720 eV were reflected to HOMO and LUMO energy levels of MPS-linker-water-NR, respectively (Fig. 8b). The HOMOs of MPS-linker-water-NR were generally distributed on nitrogen elements of NR dye, while the LUMOs of this structure were mostly situated on rings linking to NR dye and linker of mesoporous silica. In addition, the HOMOs of MPS-linker-water-CV were distributed on chlorine, while, LUMOs were placed on rings of CV dye and Si element of mesoporous silica (Fig. 8c). The energy values of − 5.9070 and − 3.0630 eV in MPS-linker-water-CV were corresponded to the HOMOs and LUMOs, respectively. In addition, The HOMOs of MPS-linker-water-OII localized on N elements of OII dye, meanwhile, the LUMOs of this structure were distributed on S and O atoms of hydroxyl and carbones of benzene ring (Fig. 8d). The energy values of − 4.4980 and + 4.0500 eV in MPS-linker-water-OII were related to the HOMOs and LUMO energy levels, respectively. The corresponding energy gaps between HOMO and LUMO (ΔE = E_LUMO_ − E_HOMO_) for water, NR, CV, OII dyes, MPS-linker, MPS-linker-water-NR, MPS-linker -water-CV and MPS-linker-water-OII molecules were about 0.3140, 0.0670, 0.0529, 1.6500, 13.681, 1.2400, 0.4300 and 0.4480 eV, respectively. Due to the lower HOMO–LUMO energy gap value of CV dye compared to NR molecules, it can be concluded that the CV adsorbtion on the MPS-linker is occurred easier due to high reactivity of CV molecule.

#### Chemical hardness (η) and softness (σ) of compounds

The chemical reactivity is of great importance for determination of the structure reactivity. Based on the chemical reactivity, molecules can be divided into hardness (*η*) and softness (*σ*) molecules and the hard structures are less reactive compared to the soft ones. The chemical hardness is a significant physicochemical property for understanding the behavior of chemical material. The soft/hard molecules have small/large energy gap. By increasing the E_gap_ value, the chemical reactivity of component is decreased while the stability is increased. The values of *η* and *σ* can be obtained using Eqs. (3) and (4)^54,26^.

3 $$\eta = \left( {\frac{I - A}{2}} \right)$$

4 $$\sigma = \frac{1}{\eta }$$

In Eq. (3), *I* refers to the the ionization potential (-E_HOMO_). Also, *A* denotes the electron affinity (–E_LUMO_). According to above mentioned Eqs., the smaller value of hardness means more reactivity and vice versa^57,58^. The chemical hardness of NR, CV, OII, MPS-linker-water-NR, MPS-linker-water-CV, and MPS-linker-water-OII structures were about 0.0349, 0.0235, 0.8250, 0.6200, 0.2150, and 0.3080 eV, respectively. According to the results, the hardness of MPS-linker-water-CV was less than MPS-linker-water-NR, and MPS-linker-water-OII. As a result, MPS-linker-water-CV is the more soft and less stable. The chemical hardness of MPS-linker-water-NR, MPS-linker-water-CV, and MPS-linker-water-OII were about 1.2400, 0.4300 and 0.4480 eV, respectively. Therefore, it can be concluded that the chemical reactivity of MPS-linker-water-CV was more than NR and OII in the presence of MPS-linker.

## Conclusions

In this research the MPS-linker adsorbent was successfully prepared via a facile one-pot sol–gel-hydrothermal method. Different characterization analyses such as FESEM, TEM, FTIR, L-XRD, BET/BJH and TGA were utilized to investigate the physicochemical properties of fabricated adsorbent. The adsorption performance of produced MPS-linker was studied in removal of natural red and crystal violet dyes. The influence of initial adsorbent amount was evaluated on removal efficiency and adsorbtion capacities. The results obtained after linear fitting of the Langmuir and Freundlich model indicated that the Langmuir model provides a better fit to the experimental data. The molecular dynamics simulations were investigated for adsorption of both cationic (NR and CV) and anionic (OII) dyes and the results were compared. The optimization of structures were carried out by the Dmol3 module. The outcomes demonstrated the adsorption ability of functionalized silica for both anionic and cathionic dyes in water environment. The chemical reactivity, global hardness and softness were measured from frontier molecular orbitals. Due to less HOMO–LUMO, E_gap_ of CV dye on MPS-linker adsorbent, the chemical reactivity and softness of CV dye were higher compared to other dyes. The results predicted the great potential of MPS-linker adsorbent for the CV removal compared to NR and OII dyes.
